# Characteristics of responders to atropine 0.01% as treatment in Asian myopic children

**DOI:** 10.1038/s41598-022-10978-3

**Published:** 2022-05-05

**Authors:** Lung-Chi Lee, Meng-Wei Hsieh, Yi-Hao Chen, Po-Liang Chen, Ke-Hung Chien

**Affiliations:** 1 Department of Ophthalmology, Tri-Service General Hospital, National Defense Medical Center, No. 325, Sec. 2, Cheng-gong Rd., Neihu Dist., Taipei, 114 Taiwan, ROC; 2 Department of Ophthalmology, Taoyuan Armed Forces General Hospital, Taoyuan, Taiwan, ROC; 3 National Defense Medical Center, Taipei, Taiwan, ROC; 4 Hau-Ming Eye Clinic Center, No. 199, Zhongxing Rd., Xizhi Dist., New Taipei City, 221 Taiwan, ROC

**Keywords:** Risk factors, Paediatric research, Outcomes research

## Abstract

Recently, low-concentration atropine (0.01%) has gained increased attention in controlling myopia progression with satisfying effects and minimal side effects. However, studies concerning responders to 0.01% atropine are limited. This retrospective observational cohort study aimed to determine the responder characteristics of 0.01% atropine in Asian children. One hundred forty children (aged between 3 and 15 years) receiving 0.01% atropine were analyzed for the factors influencing annual spherical equivalent changes (SE). The mean age was 9.13 (2.6) years, the mean baseline SE was − 1.56 (1.52) diopters (D), and the mean annual SE change was − 0.52 (0.49) D. A 58.63% responder rate (146/249) of myopic control was achieved with 0.01% atropine in our entire cohort under the criteria of less than 0.5 D of myopic progression annually. The subjects were stratified into 4 subgroups based on a cut-off point of baseline SE of − 1.5 D and baseline age of 9 years. The responder rate differed significantly with the highest being the youngest with the lowest myopia subgroups. Our results demonstrated that children with myopia better than − 1.5 D and younger than 9 years had the highest potential to achieve successful myopic control under 0.01% atropine therapy.

## Introduction

Myopia is one of the most prevalent ocular disorders in the modern world. Although the prevalence differs according to geographic region, ethnicity, cultural influence, age, and other factors, the figures are alarming^1,2^. In the United States, among children aged between 12 and 17 years, the reported prevalence of myopia was 33.9% from 1999 to 2004^3^. In urbanized countries in Asia, such as Taiwan, the prevalence of myopia in 17-year-olds exceeded 70% in 1983 and continued to increase in the subsequent years of the survey^4^. Moreover, early-onset myopia was found to be a significant risk factor for developing high myopia^5^. In younger children, the prevalence of myopia in children aged 7 has increased from 5.8% in 1983 to 21.0% in 2000 in Taiwan^4^. Among these children, up to 80–90% have myopia when finishing high school, and nearly 20% of these children are diagnosed with high myopia^4,6,7^. Therefore, epidemics of myopia and high myopia exist in these regions^8^, and with the significant economic burden associated with fighting high myopia-related complications^9,10^, early prevention and intervention are paramount among children in this age range.

Different interventions have been used for myopia control in children. However, pharmacologic modalities, namely, atropine eye drops, are currently the most effective treatment^11,12^. Topical atropine at a 1% concentration was shown to slow myopia progression and axial elongation in Asian children^13^. However, despite having satisfactory effect of myopia control, adverse effects, such as photophobia and accommodative paresis, can be problematic in children associated with poor treatment results due to inadequate adherence to the therapy^14,15^. Hence, a lower concentration of atropine has been used to meet the balance between treatment efficacy and minimal side effects^16^.

Recently, 0.01% atropine has gained increased attention among pediatric ophthalmologists for treating childhood myopia^17^. In the Atropine for the Treatment of Myopia (ATOM) 2 study, 0.01% atropine, compared with the placebo in the ATOM1 study^13^, demonstrated a 59% rate of slowing myopia progression in 2 years of treatment with minimal side effects compared with higher concentrations (0.1% and 0.5% atropine)^15,18,19^. In the Low-Concentration Atropine for Myopia Progression (LAMP) study, 0.01% atropine, despite its inferior efficacy compared with 0.025% and 0.05% atropine, still showed a 27% and 27.7% reduction in myopia progression 1 and 2 years after treatment, respectively^20,21^. Other studies have also demonstrated the efficacy of 0.01% atropine in treating childhood myopia^22–34^. However, limited information is available concerning who would benefit most from 0.01% atropine treatment in controlling myopia progression. This study aimed to determine the characteristics of responders to 0.01% atropine in Asian children aged between 3 and 15 years.

## Methods

This retrospective study was conducted to collate data on myopic children treated with 0.01% atropine at Tri-Service General Hospital from January 2011 to December 2016. The study protocol and supporting documents were reviewed and approved by the institutional review board (No: A202105131) of the Tri-Service General Hospital, Taipei, Taiwan. Informed consents were waived due to the retrospective nature of this study according to the institutional review board. The study followed the Good Clinical Practice guidelines of Taiwan and was performed in accordance with the Declaration of Helsinki (1964) and later revisions.

Patients were eligible for inclusion if they were aged between 3 and 15 years at the initial visit; if they were diagnosed with myopia in each eye when their cycloplegic spherical equivalent (SE) was equal to or worse than − 0.25 diopters (D) with best-corrected visual acuity (BCVA) better than 0.1 logarithm of the minimum angle of resolution (logMAR) (Snellen equivalents: 20/25); and if they were documented with having a progression of myopia before any treatment and then treated with 0.01% atropine once per night for myopia control. If both eyes met the above inclusion criteria, both eyes were chosen for analysis. Patients were excluded from the study if they had other concurrent ocular diseases, such as strabismus, cataract, or other ocular diseases that could hinder vision development; if they had a previous history of amblyopia treatment before this study, such as wearing spectacles, atropine penalization, or patching; if they had a follow-up duration of less than 1 year; or if they had ever received alternative myopia treatments, such as orthokeratology or other modalities.

Detailed information from the medical records during each follow-up visit was analyzed. The data from the charts regarding the BCVA, cycloplegic refractive error including spheres and cylinders (dioptric power and axis), concentration and dosage of atropine, and follow-up duration were collected for analysis. Cycloplegic refractive errors were measured routinely in our clinic using a Tonoref III autorefractor (Nidek Co., Ltd., Japan) 30 min after the instillation of 3 drops of 1% cyclopentolate eye drops every 5 min. The readings from the autorefractor were averaged and confirmed using retinoscopy examinations performed by the same ophthalmologist (Chen); he also ensured that the complete cycloplegic effect was achieved before each examination. All the included children were prescribed full-correction single-vision spectacle lenses as needed, and the appropriate changes to the spectacle prescriptions were made at each follow-up by Chen.

During the study period, if the children had their atropine regimen shifted to another concentration, such as 0.1% or 0.3%, these patients were excluded from the analysis entirely. Treatment adherence was routinely ensured with the parents during every clinic visit using the EyePass passport (handbook issued by the Taiwan’s government for myopic prevention policy) and documented in the medical records.

A mean annual increase in SE less than 0.5 D gain (i.e., better control of myopia with less than 0.5 D progression) or more was chosen to stratify the response to 0.01% atropine. Subjects included were further sub-analyzed in 2 separate groups according to their treatment responses; the responder group was defined as less than 0.5 D mean annual myopia gain and the non-responder group was defined as mean annual myopia gain greater than or equal to 0.5 D.

The characteristics of both groups were further analyzed using SPSS software version 16.0 for Windows (SPSS Inc., Chicago, IL, USA). The data for each group were presented as means (standard deviation, SD). We conducted Student’s t-test to compare the patient’s characteristics under myopia treatment, logistic regression analysis to examine predictive factors for treatment responders, and receiver operating characteristic (ROC) curve analysis to identify cut-off points between the stratified groups. *p* < 0.05 was considered statistically significant.

## Results

One hundred forty children (70 boys and 70 girls) with at least one myopic eye met the inclusion criteria. In total, 249 eyes were analyzed in this study. All the patients chosen for the current study had received 0.01% atropine for at least 12 months, and the mean (SD) follow-up period was 2.54 (1.1) years. The mean baseline age (SD) was 9.13 (2.6) years, the mean baseline SE was − 1.56 D (1.52), and the overall mean annual SE change was − 0.52 D (0.49).

To evaluate the factors and characteristics regarding the treatment response in these patients, we divided the patients into two groups: 140 eyes were in the responder group and 109 eyes were in the non-responder group. Overall, children received 0.01% atropine for an average of 2.68 years (1.18) in the responder group and for an average of 2.36 years (0.97) in the non-responder group (*p* = 0.090). The mean (SD) baseline age was 7.97 (2.29) years in the responder group and 10.61 (2.23) years in the non-responder group (Table 1). The children in the responder group were significantly younger than those in the non-responder group (*p* < 0.0001). Next, we further analyzed the refractive errors between the responder and non-responder groups. The mean baseline SE was − 0.79 D (0.86) in the responder group and − 2.55 D (1.61) in the non-responder group (Table 1). The baseline SE was significantly different between the two groups (*p* < 0.001). At the last visit from the chart review, the results between both groups also revealed statistical significance. The total SE change was − 0.59 D (0.71) in the responder group in contrast to − 1.61 D (0.84) in the non-responder group (*p* < 0.001). The SE increased annually by − 0.41 D (0.40) in the responder group and by − 1.59 D (0.74) in the non-responder group (*p* < 0.001).

**Table 1:** Demographic characteristics of children in the study.

	Entire cohort	Responder group	Non-responder group	*p*
No. of subjects (eyes)	140 (249)	85 (140)	55 (109)	
Male (eyes)	70 (117)	39 (65)	31 (52)	0.136
Female (eyes)	70 (132)	46 (86)	24 (46)	0.067
Baseline age (years) (SD)	9.13 (2.6)	7.97 (2.29)	10.61 (2.23)	< 0.001
Baseline SE (D) (SD)	− 1.56 (1.52)	− 0.79 (0.86)	− 2.55 (1.61)	< 0.001
Total SE change (D) (SD)	− 0.93 (0.82)	− 0.59 (0.71)	− 1.61 (0.84)	< 0.001
Annual SE change (D) (SD)	− 0.52 (0.49)	− 0.41 (0.40)	− 1.59 (0.74)	< 0.001
Treatment period (years) (SD)	2.54 (1.1)	2.68 (1.18)	2.36 (0.97)	0.090

The *p* values compare the responder and non-responder groups.

*D* diopter, *SE* spherical equivalent, *SD* standard deviation.

Next, we investigated the predictive factors leading to the difference in the treatment response with 0.01% atropine. Factors such as the sex (not shown), baseline age, baseline SE, astigmatism degree (not shown), treatment duration, and total SE change were used for logistic regression analysis. In the logistic regression analysis, the only factors leading to a treatment response were the baseline SE and baseline age (*p* < 0.001) (Table 2).

**Table 2:** Factors influencing the 0.01% atropine treatment results.

	Unstandardized coefficients		Standardized coefficients	t	Sig.
	B	Std. Error	Beta		
Baseline age (years)	0.002	0.007	0.008	0.213	< 0.0001
Baseline SE (D)	− 0.022	0.014	− 0.68	− 1.631	< 0.0001
Total SE changes (D)	− 0.076	0.032	− 1.25	− 2.410	0.831
Treatment period (years)	− 0.054	0.014	− 0.188	− 3.735	0.104

The data were obtained via logistic regression.

*D* diopter, *SE* spherical equivalent, *SD* standard deviation.

**Figure 1:**
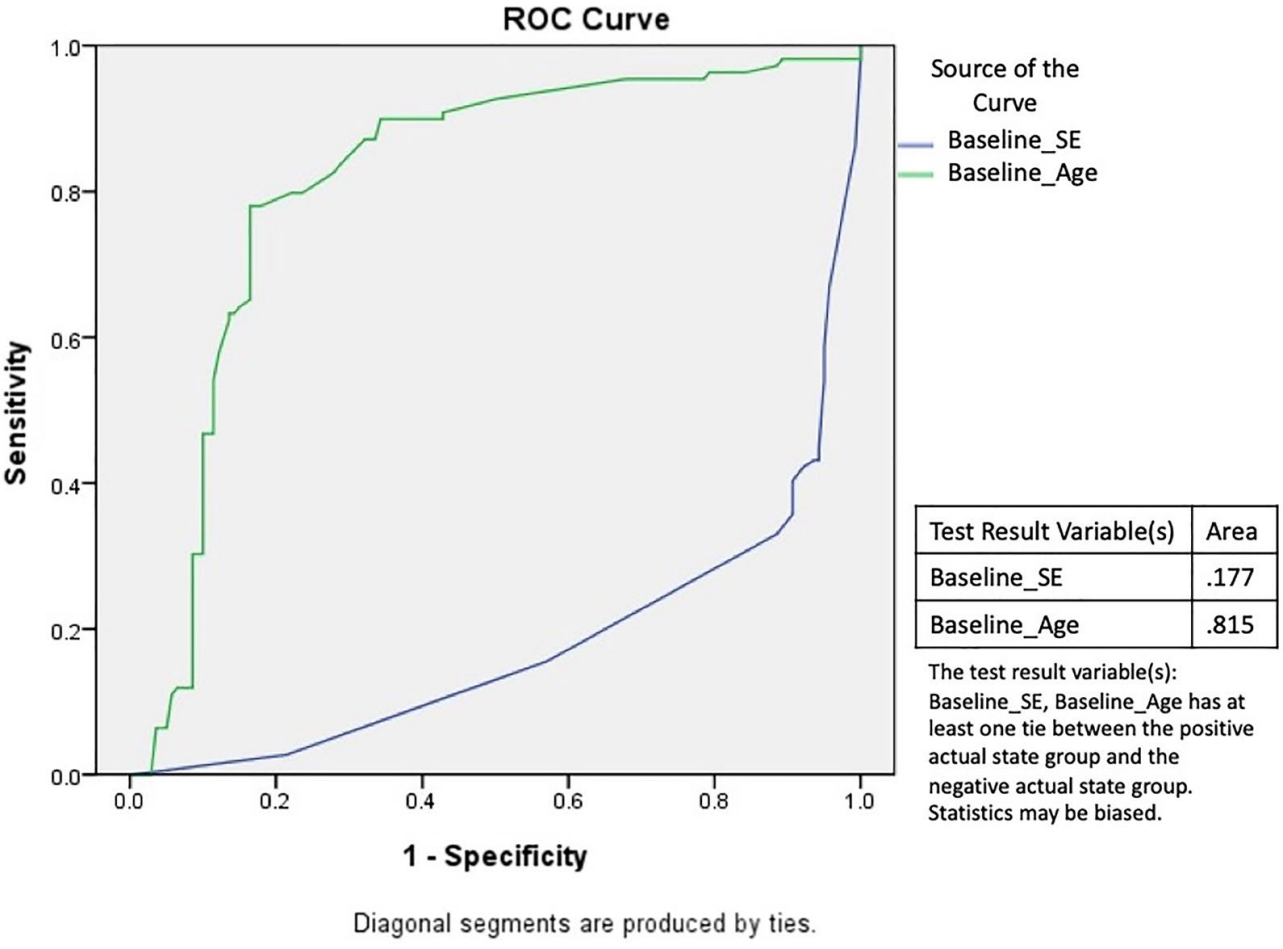
Receiver operating characteristic (ROC) curve analysis of the treatment results based on the baseline spherical equivalent (SE) and baseline age. The cut-off point of the baseline SE was − 1.25 D and that of the baseline age was 8.9 years to achieve the best treatment response.

According to the above results, we further identified the cut-off points that led to a better treatment response for the baseline SE and baseline age using ROC curve analysis; the best cut-off point for the baseline SE was − 1.25 D and that for the baseline age was 8.9 years (Fig. 1). Next, we then further stratified our subjects into 4 groups based on a baseline SE of − 1.5 D and a baseline age of 9 years using the cut-off values derived from Fig. 1. Children stratified in group 1 with a baseline SE better than − 1.5 D (less myopia at baseline) and age younger than 9 years had the highest responder rate (90.48%). When children were older than 9 years, the responder rate decreased to 30.95% in children whose baseline SE was better than − 1.5 D (group 3) and 15.38% in children whose baseline SE was worse than − 1.5 D (group 4). Notably, a higher responder rate was found in children with baseline SE values worse than − 1.5 D and in the younger group (group 2, 56.25%) compared with those in the older group (group 4, 15.38%). Children younger than 9 years had a significantly higher responder rate than older children (86.62% vs. 21.50%). Additionally, children with less myopia at baseline had a higher responder rate than those with higher myopia (75.60% vs. 23.46%). Overall, considering an annual myopia gain of less than 0.5 D as a treatment responder, our entire cohort had a 58.63% success rate of myopic control under 0.01% atropine treatment (Table 3).

**Table 3:** Responder rates of 0.01% atropine treatment in subgroups.

	Baseline age ≤ 9	Baseline age > 9	
Baseline SE > − 1.5 D	(Group 1) 114/126 = 90.48%	(Group 3) 13/42 = 30.95%	127/168 = 75.60%
Baseline SE ≤ − 1.5 D	(Group 2) 9/16 = 56.25%	(Group 4) 10/65 = 15.38%	19/81 = 23.46%
	123/142 = 86.62%	23/107 = 21.50%	146/249 = 58.63%

*D* diopter, *SE* spherical equivalent.

**Figure 2:**
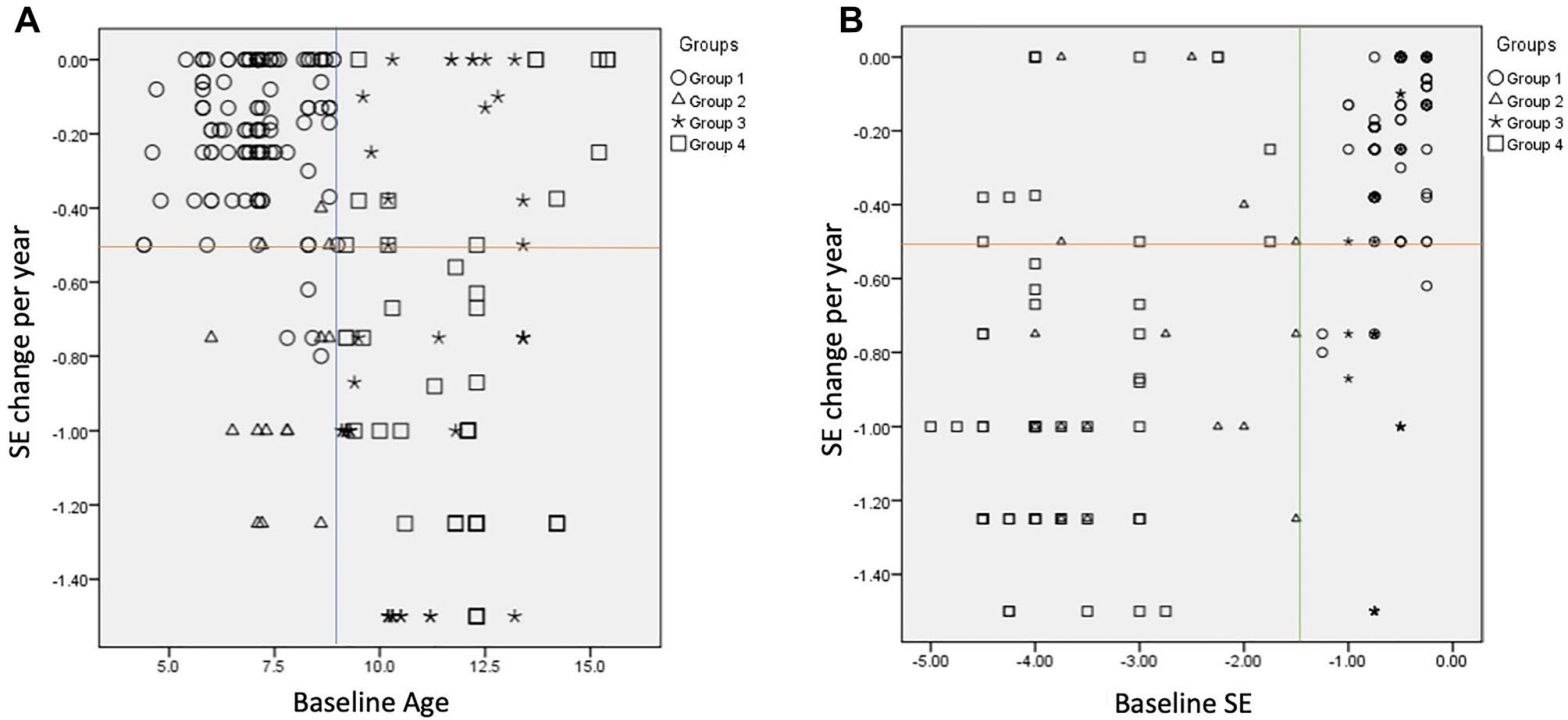
Scatter plots of all the study participants based on the cut-off points of a baseline age of 9 years (blue vertical line) and baseline spherical equivalent (SE) of − 1.5 D (green vertical line). (**A**) Plot of baseline age versus annual SE changes. (**B**) Plot of baseline SE versus annual SE changes. The orange line signifies the threshold of responders vs. non-responders for a half-diopter change annually.

Using the cut-off points for the baseline SE (− 1.5 D) and baseline age (9 years) in the analysis, we plotted our study subjects according to the 4 stratified groups with their annual SE changes against the baseline age (Fig. 2A) and baseline SE (Fig. 2B). Significant differences were found in the distribution of our study subjects. In Fig. 2A, the distribution of group 1 (baseline SE > − 1.5 D and age ≤ 9 years old) was more concentrated in the left-upper quadrant. In Fig. 2B, the distribution of subjects with lower myopia at baseline (group 1 and group 3) was more concentrated in the right-upper corner. Subjects with less myopia at baseline (group 1 and group 3) and with age younger than 9 years (group 1 and group 2) were also relatively concentrated in the upper part of the graph in Fig. 2A,B, respectively, which mirrored our results.

## Discussion

Based on our subjects in this cohort study, children with myopia less than − 1.5 D and who were younger than 9 years at baseline (group 1) had the highest responder rate (defined as the percentage of patients with less than 0.50 D annual myopia gain) when treated with 0.01% atropine. In this patient group (group 1), the responder rate of 0.01% atropine for myopic control was as high as 90.48%, which is much higher than the overall responder rate in our series (58.63%) and higher than prior reports with a more generalized group (unstratified by the baseline age or SE) (82% in ATOM2 and 72.6% in LAMP2 after one year of 0.01% atropine treatment)^15,18,20,21^. Other studies have reported various responder rates, from 22^21^ to 98%^28^ (Supplementary Table S1).

Additionally, when the mean annual myopia gain was used as the parameter to evaluate the efficacy of 0.01% atropine in controlling myopia, different numbers of mean annual myopia progression have been reported in the literature, ranging from 0.26 D (decrease in myopia) in a prospective study in 1998^33^ to − 0.84 D in a recent Korean study^29^ (Supplementary Table S1). In the famous phase 1 study of ATOM2, children treated with 0.01% atropine had a − 0.43 D and − 0.49 D myopia gain from baseline over the first and second years, respectively^15^. Additionally, in the phase 3 study of ATOM2, all the groups that exhibited myopia progression (> 0.5D) in the wash-out period (phase 2)^19^ were retreated with 0.01% atropine^18^. The results showed that children originally treated with 0.01% atropine in the phase 1 study showed a − 0.42 D and − 0.44 D myopia gain in the fourth and fifth years of the phase 3 study, respectively^18^. In the LAMP1 and LAMP2 studies, children were also treated with 0.01% atropine and exhibited a − 0.59 D and − 0.48 D of myopia gain consecutively in the first and second study years^20,21^. Our results showed a similar overall mean annual myopia gain (− 0.52 D) to that of these previous studies.

Interestingly, when reviewing the baseline characteristics, a large difference was found in the baseline SE among these studies (Supplementary Table S1). For example, in the ATOM2 and LAMP1 studies, the participants had much higher myopia on average (− 4.5 D and − 3.77 D in the 0.01% atropine group of the ATOM2 16 and LAMP1^20^ studies, respectively) than that in our study (− 1.56 D). The higher baseline SE of their participants might reflect the myopia range chosen for subject recruitment, which was considerably higher than ours (compared and listed in Supplementary Table S1). Furthermore, because of the different inclusion age ranges, the average baseline age differed among studies (Supplementary Table S1). Hence, comparing these results while discussing the efficacy of 0.01% atropine treatment in children, clinicians should interpret with caution because of the diversity of these study designs and their participants’ basic characteristics^35^.

Among our study subjects a low negative relationship was found between the baseline age and annual SE change (Pearson’s correlation coefficient: − 0.284; *p* = 0.008) and a high negative relationship was found between the baseline age and baseline SE (Pearson’s correlation coefficient: − 0.554; *p* < 0.001). In other words, in our series, the younger were the children, the lower was the baseline myopia and the lesser was the amount of annual myopia progression. When subgrouping our subjects according to the baseline age and baseline SE, our results revealed differences in the responder rate between the groups (Table 3). Younger children with less myopia at baseline (group 1) responded well in our series. A prospective study conducted in Spain with similar basic characteristics reported an excellent result with a 98% responder rate (< 0.5 D progression) when treated with 0.01% atropine^28^. The author concluded that treatment initiated in low baseline myopia (− 1.0 D) and at early ages (10–11 years) showed a positive response to treatment^28^. In another retrospective study conducted in the United States with higher myopia (− 2.0 D) on average, the author also concluded that 0.01% atropine was most effective in low initial myopia^22^.

By contrast, in our series, older children (age older than 9 years) with higher myopia (SE < − 1.5 D) at baseline had the lowest responder rate (15.38%); in other words, most of the patients who had undergone 0.01% atropine treatment had an annual myopia progression over 0.50 D (considered rapid progressor) in this stratified subgroup (group 4). In a recent randomized controlled trial conducted in India, progressors were older (11 (2) years) with higher myopia (− 3.12 (1.07) D) at baseline^32^. In a retrospective case–control study conducted in Italy, progressors had a mean SE of − 3.24 (1.32) D and a mean age of 9.0 (2.34) years, and none of these participants were young children (age < 6 years)^23^.

However, when analyzing the influence of the baseline age and baseline SE on the effect of atropine treatment in myopia control, the results in the literature are inconclusive with sometimes contradictory results. In the ATOM1 study on risk factors for progressive myopia with 1% atropine treatment, 12.1% of participants were considered progressors and tended to be younger (8.5 (1.4) years) with higher baseline myopia (− 3.66 (1.3) D)^36^. In the ATOM2 study, multivariate analysis revealed that younger children (9.1 (1.3) years) with higher concentrations of atropine (0.5% and 0.1%) administered in phase 1 had higher rebound in phase 2 and were more likely to be re-treated in phase 3; these children were less myopic (− 4.34 (1.64) D vs. − 4.70 (1.51) D, *p* = 0.031) with a shorter axial length (25.05 (0.91) mm vs. 25.30 (0.86) mm, *p* = 0.008) at baseline than the untreated group^18^. In another post hoc analysis of the LAMP1 study, age-dependent and concentration-dependent treatment effects were observed^37^. The authors concluded that the younger the child was, the higher the concentration of atropine treatment should be to achieve a comparable effect on myopia control and that the baseline SE was not a factor influencing myopia progression^37^. Another retrospective study conducted in Shanghai reported a similar study design to ours but different results; the author found a significantly lower baseline SE in the progressor group (− 3.12 (2.03) D vs. − 4.26 (2.96) D) than in the non-progressor group and that with higher myopia (every 1.0 D gain) at baseline, the risk of myopia progression was 14% lower^26^. On the contrary, in another study examining the long-term results of 0.05% atropine in Taiwanese children, the author found that a lower baseline SE was significantly associated with less myopia progression^38^.

In addition to the inherent differences in the region, ethnicity, and study designs among these studies (Supplementary Table S1), the perplexing conclusions were a possible continuum of the basic characteristic differences, in both the baseline age and baseline SE, among these studies, which would cause a huge conundrum when comparing these results. The major difference between the abovementioned studies and our present study is the low baseline SE (− 1.56 (1.52) D) and 2 clustered ranges of baseline SE in our subjects (Fig. 2B). This specific distribution could lead to our current conclusion that low initial myopia would have an excellent response to 0.01% atropine treatment, contradicting the results of the ATOM1 and LAMP1 studies with higher myopia at baseline than ours. However, in another prospective study in Taiwan using 0.025% atropine for myopia prevention in premyopic children (baseline age 7.6 (1.7) years and baseline SE − 0.31(0.45) D), the effect was significant (− 0.14 (0.24) D in 0.025% atropine group vs. − 0.58 (0.34) D in control group, *p* < 0.0001); the author also concluded that it is important for school-aged children to maintain their SE in the hyperopic or premyopic status to prevent a further myopia shift and onset^39^. Hence, the responder characteristics can be very different in patients with different baseline SEs. Additionally, children who had undergone these clinical trials grew old during the studies, and usually, their myopia also progressed alongside it. Hence, in a cohort observational study or a short-duration prospective study, the results of children who were older or with higher myopia were actually those with myopia (lesser) at an earlier age. Therefore, patients with a younger age and less myopia (group 1) can either grow into older children with better controlled myopia (no or slow myopia progression, group 3) or uncontrolled myopia (continuous or rapid myopia progression, group 4). For patients with higher myopia at a younger age (group 2), they had passed the threshold of premyopia at an earlier stage and had an alarming possibility of progressing to high myopia in the future (group 4)^5^. Following this rationale, clinicians should provide adequate treatment regimens (different concentrations of atropine) according to the basic characteristics of their patients.

Our current study has some limitations. First, as a retrospective study in nature, some information may also be limited or biased because of our inclusion and exclusion criteria. The exposure of other factors that would influence myopia progression, such as outdoor activities, may not be controlled in a retrospective design. Second, the lack of a control group limits the ability of a direct comparison. Third, because of the nature of a retrospective study, not all subjects have complete axial length data available throughout our study period. Hence, for the completeness of the data collected, we did not analyze the axial length changes. However, even though axial length elongation has been proposed in the literature as the main mechanism of axial myopia progression and the standard way to monitor myopia progression^35^. Our study resembled a real-life clinical scenario of a busy general ophthalmology clinic, which relies heavily on skillful and accurate cycloplegic refraction for myopia follow-up and prescribing full-correction single-vision spectacle lenses and usually lacks additional machinery for routine axial length measurement.

In conclusion, our results demonstrated that 0.01% atropine showed good results in myopic control. Children with myopic refraction better than − 1.5 D and who were younger than 9 years had the highest potential to achieve myopic control under 0.01% atropine therapy in our cohort. Conversely, because of the high progression rate in children older than 9 years and initial myopia worse than − 1.5 D, other therapy choices, such as shifting to higher concentrations of atropine or using orthokeratology lenses alone or in combination with 0.01% atropine, should be considered. However, due to the diversity of study designs, there were contradictory results in the literature for the characteristics of responders to 0.01% atropine in treating myopic children. Further international and multicenter long-term cohort studies may need to be employed in different populations and clinical settings to verify the risk factors for progression.

## Supplementary Information

Supplementary Information.

## Data Availability

The data presented in this study are available on request from the corresponding author. The data are not publicly available due to the restriction of the Institute of Institutional Review Board of the Tri-Service General Hospital, Taipei, Taiwan.
