# Supplementary material for: Characteristics of responders to atropine 0.01% as treatment in Asian myopic children
Source: Sci Rep. 2022 May 5;12:7380. doi: 10.1038/s41598-022-10978-3 (PMC9072680; doi:10.1038/s41598-022-10978-3)
Supplement: Supplementary file 1 — Supplementary Information. [file 41598_2022_10978_MOESM1_ESM.pdf]

Supplementary Table S1. Summary of clinical studies involving 0.01% atropine

Study design and Inclusion criteria							Results					
Study, year	Country / Ethnic	Study design	Age range (year)	Myopia range (D)	Duration[a] (year (SD))	Treatment group (participant size)	Baseline age (year (SD))	Baseline spherical equivalent (D (SD))	Myopia progression[b] (D (SD))	Baseline axial length (mm (SD))	Axial elongation[b] (mm (SD))	Responder rate[d]
Lee et al. (current study), 2021	Taiwan	single-center, retrospective cohort study	3-15	At least -0.25	2.54 (1.1)	0.01% atropine (140)	9.13 (2.6)	-1.56 (1.52)	-0.52 (0.49)	N/A	N/A	71.5% (<1.0D);<br>58.63% (<0.5D)<br>(group1) 90.48%<br>(group2) 56.25%<br>(group3) 30.95%<br>(group4) 15.38%
						Responder (85)	7.97 (2.29)	-0.79 (0.86)	-0.41 (0.40)			
						Non-responder (55)	10.61 (2.23)	-2.55 (1.61)	-1.59 (0.74)			
Hu[33], 1998	Shanghai, China	single-center, RCT	9-18	-0.50 to -3.00	1	1% atropine (192)	13.5	-2.14	0.79	N/A	N/A	96.3% (<0.5D)
						0.1% atropine (183)		-2.25	0.43			90.7% (<0.5D)
						0.01% atropine (161)		-2.24	0.26			75.2% (<0.5D)
						control (536, contra-eye)		-2.23	-0.42			60.9% (<0.5D)
Chia et al.[15] (ATOM2), 2012	Singapore	single-center, RCT	6-12	At least -2.00	2<br>(phase 1: 1st and 2nd year result)	0.5% atropine (139)	9.7 (1.5)	-4.3 (1.8)	- 0.17 (0.47) at 12 mo<br>- 0.30 (0.60) at 24 mo	25.2 (0.9)	0.11 (0.17) at 12 mo<br>0.27 (0.25) at 24 mo	63% (<0.5D);<br>82% (<1.0D)<br>(at 24 mo)
						0.1% atropine (141)	9.7 (1.6)	-4.5 (1.4)	-0.31 (0.50) at 12 mo<br>-0.38 (0.60) at 24 mo	25.2 (0.8)	0.13 (0.18) at 12 mo<br>0.28 (0.27) at 24 mo	58% (<0.5D);<br>82% (<1.0D)<br>(at 24 mo)
						0.01% atropine (75)	9.5 (1.5)	-4.5 (1.5)	-0.43 (0.52) at 12 mo<br>-0.49 (0.63) at 24 mo	25.1 (1.0)	0.24 (0.19) at 12 mo<br>0.41 (0.32) at 24 mo	50% (<0.5D);<br>82% (<1.0D)<br>(at 24 mo)
Clark et al.[22], 2015	United States / diverse	single-center, retrospective case-control study	6-15	-0.25 to -8.00	1	0.01% atropine (32)	10.2 (2.2)	-2.0 (1.6)	-0.1 (0.6)	N/A	N/A	91% (<1.0D)
						control (28)	10.2 (2.2)	-2.0 (1.5)	-0.6 (0.4)			86% (<1.0D)
Chia et al.[18] (ATOM2), 2016	Singapore	single-center, RCT	6-12	At least -2.00	2<br>(phase 3: add 4th and 5th year result)	Original 0.5% atropine -> cease -> 0.01% atropine (93)	9.2 (1.4)<br>(at baseline)	-5.83(1.78)<br>(at 36 mo)	-0.42 (0.51) at 36-48 mo<br>-0.49 (0.56) at 48-60 mo	25.87 (1.06)<br>(at 36 mo)	0.17 (0.16) at 36-48 mo<br>0.12 (0.13) at 48-60 mo	N/A
						Original 0.1% atropine -> cease -> 0.01% atropine (82)	9.0 (1.3)<br>(at baseline)	-5.78 (1.28)<br>(at 36 mo)	-0.38 (0.50) at 36-48 mo<br>-0.52 (0.44) at 48-60 mo	25.76 (0.85)<br>(at 36 mo)	0.15 (0.15) at 36-48 mo<br>0.12 (0.12) at 48-60 mo	
						Original 0.01% atropine -> cease -> 0.01% atropine (17)	8.6 (1.1)<br>(at baseline)	-5.78 (1.28)<br>(at 36 mo)	-0.42 (0.47) at 36-48 mo<br>-0.44 (0.48) at 48-60 mo	25.89 (0.92)<br>(at 36 mo)	0.17 (0.15) at 36-48 mo<br>0.15 (0.11) at 48-60 mo	
						Original 0.5% / 0.1% / 0.01% atropine -> cease -> control (43 / 57 / 53)	10.9 (1.0) /<br>10.7 (1.2) /<br>10.0 (1.3)<br>(at baseline)	-5.05 (1.54) /<br>-5.18 (1.36) /<br>-5.27 (1.64)<br>(at 36 mo)	-0.38 (0.37) / -0.36 (0.42) / -0.30 (0.39)<br>(at 36-48 mo)<br><br>-0.32 (0.34) / -0.36 (0.42) / -0.34 (0.38)<br>(at 48-60 mo)	25.56 (0.85) /<br>25.66 (0.85) /<br>25.82 (1.10)<br>(at 36 mo)	0.10 (0.09) / 0.13 (0.10) / 0.08 (0.09)<br>(at 36-48 mo)<br><br>0.08 (0.10) / 0.07 (0.09) / 0.06 (0.08)<br>(at 48-60 mo)	
Diaz-Llopis et al.[28], 2018	Spain	single-center, RCT	9-12	-0.50 to -2.00	5	0.01% atropine (100)	10.4 (2.5)	-1.1 (0.50)	-0.14 (0.35)	N/A	N/A	98% (<0.5D)
						control (100)	10.1 (2.2)	-1.2 (0.40)	-0.65 (0.54)			79% (<0.5D)

Moon et al.[29], 2018	Korea	single-center, retrospective cohort study	5-14	Below -6.00	1 (0.1)	0.01% atropine (89)	8.0 (2.2)	-3.84 (2.47)	-0.84 (0.86)	24.86 (1.22)	0.44 (0.32)	61.8% (<1.0D)
					0.9 (0.1)	0.025% atropine (63)	8.4 (2.1)	-3.97 (1.65)	-0.56 (0.86)	24.66 (0.93)	0.30 (0.24)	65.1% (<1.0D)
					1.1 (0.2)	0.05% atropine (133)	8.1 (2.1)	-3.94 (2.76)	-0.23 (0.67)	24.91 (1.43)	0.23 (0.25)	93.2% (<1.0D)
Larkin et al.[27], 2019	United States / diverse	multicenter, retrospective case-control study	6-15	-0.25 to -8.00	2	0.01% atropine (100)	9	- 3.1 (1.9)	- 0.2 (0.8) at 12 mo<br>-0.3 (1.1) at 24 mo	N/A	N/A	78% (<0.75D) (at 12 mo); 63% (<0.75D) (at 24 mo)
						control (98)		- 2.8 (1.6)	- 0.6 (0.4) at 12 mo<br>-1.2 (0.7) at 24 mo			59% (<0.75D) (at 12 mo); 20% (<0.75D) (at 24 mo)
Joachimsen et al.[24], 2019	Germany	single-center, retrospective cohort study	6-17	N/A	1	0.01% atropine (56)	11	- 3.85 (1.88)	- 0.40 (0.49)	N/A	N/A	N/A
Sacchi et al.[23], 2019	Milan, Italy	single-center, retrospective case-control study	5-16	N/A	1	0.01% atropine (52)	9.7 (2.3)	-3.0 (2.23)	-0.54 (0.61)	N/A	N/A	79% (<0.5D)
						control (50)	12.1 (2.9)	-2.63 (2.68)	-0.80 (0.38)			N/A
Yam et al.[20] (LAMP1), 2019	Hong Kong, China	single-center, RCT	4-12	At least -1.00	1 (phase 1: 1st year result)	0.05% atropine (102)	8.45 (1.81)	-3.98 (1.69)	-0.27 (0.61)	24.85 (0.90)	0.20 (0.25)	69.6% (<0.5D); 84.8% (<1.0D)
						0.025% atropine (91)	8.54 (1.71)	-3.71 (1.85)	-0.46 (0.45)	24.86 (0.95)	0.29 (0.20)	51.6% (<0.5D); 87.4% (<1.0D)
						0.01% atropine (97)	8.23 (1.83)	-3.77 (1.85)	-0.59 (0.61)	24.70 (0.99)	0.36 (0.29)	43.8% (<0.5D); 72.5% (<1.0D)
						control (93)	8.42 (1.72)	-3.85 (1.95)	-0.81 (0.53)	24.82 (0.97)	0.41 (0.22)	24.2% (<0.5D); 62.9% (<1.0D)
Yam et al.[21] (LAMP2), 2020	Hong Kong, China	single-center, RCT	4-12	At least -1.00	2 (phase 2: 1st and 2nd year result)	0.05% atropine (93)	8.32 (1.71) (at baseline)	-3.93 (1.63) (at baseline)	-0.30 (0.44) (at 12-24 mo)	24.88 (0.91) (at baseline)	0.18 (0.16) (at 12-24 mo)	52.7 % (<0.5D)
						0.025% atropine (86)	8.48 (1.69) (at baseline)	-3.88 (1.83) (at baseline)	-0.39 (0.48) (at 12-24 mo)	24.94 (0.9) (at baseline)	0.22 (0.18) (at 12-24 mo)	32.0% (<0.5D)
						0.01% atropine (91)	8.35 (1.8) (at baseline)	-3.99 (1.94) (at baseline)	-0.48 (0.44) (at 12-24 mo)	24.78 (1.02) (at baseline)	0.25 (0.18) (at 12-24 mo)	22.0% (<0.5D)
						switch-over 0.05% atropine (80)	8.41 (1.87) (at baseline)	-4.31 (1.96) (at baseline)	-0.18 (0.49) (at 12-24 mo)	24.96 (1.02) (at baseline)	0.15 (0.18) (at 12-24 mo)	27.5% (<0.5D)
Fu et al.[30], 2020	Zhengzhou, China	single-center, RCT	6-14	-1.25 to -6.00	1	0.02% atropine (117)	9.4 (1.8)	-2.76 (1.47)	-0.38 (0.35)	24.60 (0.72)	0.30 (0.21)	50.2% (<0.5D); 83.3% (<1.0D)
						0.01% atropine (119)	9.3 (1.9)	-2.70 (1.64)	-0.47 (0.45)	24.58 (0.74)	0.37 (0.22)	45.1% (<0.5D); 79.7% (<1.0D)
						control (100)	9.5 (1.4)	-2.68 (1.42)	-0.70 (0.60)	24.55 (0.71)	0.46 (0.35)	28.1% (<0.5D); 64.4% (<1.0D)
Wei et al.[31], 2020	Beijing, China	single-center, RCT	6-12	-1.00 to -6.00	1	0.01% atropine (76)	9.44 (1.80)	−2.52 (1.33)	-0.49 (0.42)	24.50 (0.76)	0.32 (0.19)	69.9% (<0.5D); 86.8% (<1.0D)
						control (83)	9.84 (1.53)	−2.64 (1.46)	-0.76 (0.50)	24.69 (0.97)	0.41 (0.19)	51.3% (<0.5D); 65.1% (<1.0D)
Zhang et al.[26], 2020	Shanghai, China	single-center, retrospective cohort study	3-14	plano to -12.00	1	0.01% atropine (133)	5.79 (2.20)	-3.92 (2.76)	-0.55 (0.57)	24.79 (1.29)	0.43 (0.52)	69.92% (<0.75D)
						Responder (186)	7.56 (2.48)	-4.26 (2.96)			0.34 (0.58)	
						Non-responder (80)	6.99 (2.19)	-3.12 (2.03)			0.62 (0.25)	

Hieda et al.[25], 2021	Japan	multicenter, RCT	6-12	-1.00 to -6.00	2	0.01% atropine (84)	8.99 (1.44)	-2.91 (0.29)	-0.69 (0.09) at 12 mo<br>-1.26 (0.09) at 24 mo	24.43 (0.17)	0.35 (0.04) at 12 mo<br>0.63 (0.04) at 24 mo	N/A
						control (84)	8.98 (1.50)	-2.98 (0.29)	-0.77 (0.1) at 12 mo<br>-1.48 (0.09) at 24 mo	24.51 (0.17)	0.39 (0.04) at 12 mo<br>0.77 (0.04) at 24 mo	
Saxena et al.[32], 2021	India	multicentric, RCT	6-14	-0.50 to -6.00	1	0.01% atropine (50)	10.6 (2.2)	-3.5 (1.3)	-0.16 (0.4)	24.62 (0.98)	0.22 (0.2)	87% (<0.5D); 64% (<0.25D)
						control (50)	10.8 (2.2)	-3.7 (1.3)	-0.35 (0.4)	24.7 (0.74)	0.28 (0.28)	62% (<0.5D); 30% (<0.25D)
Pérez-Flores et al.[34], 2021	Spain	multicentric prospective	6-14	-2.00 to -6.00	1	0.01% atropine (92)	9.76 (1.93)	-3.56 (1.12)	-0.44 (0.41)	24.57 (0.79)	0.27 (0.20)	61.9% (<0.5D)

Abbreviation: ATOM = Atropine for the Treatment of Myopia study; D = diopter; LAMP = Low-Concentration Atropine for Myopia Progression study; mo = month; N/A = not applicable, data not shown in the article; RCT = randomized clinical trial; SD = standard deviation. The data were presented as means (SD).

a: The duration of each study was listed and specified in the column.

b: Myopia progression was defined as spherical equivalent change over one year (or other duration specified within the column) or mean annual spherical equivalent change.

c: Axial elongation was defined as axial length change over one year (or other duration specified within the column) or mean annual axial length change.

d: The responder rate was defined as percentage of participant less than 1 diopter change (or less, specified within the column) within 1 year (or other duration specified within the column); data were adopted from the information provided in the articles.
